# The neuritic plaque facilitates pathological conversion of tau in an Alzheimer's disease mouse model

**DOI:** 10.1038/ncomms12082

**Published:** 2016-07-04

**Authors:** Tong Li, Kerstin E. Braunstein, Juhong Zhang, Ashley Lau, Leslie Sibener, Christopher Deeble, Philip C. Wong

**Affiliations:** 1 Department of Pathology, The Johns Hopkins University School of Medicine, 720 Rutland Avenue, Ross 558, Baltimore, Maryland 21205, USA; 2 Department of Neuroscience, The Johns Hopkins University School of Medicine, Baltimore, Maryland 21205, USA

## Abstract

A central question in Alzheimer's Disease (AD) is whether the neuritic plaque is necessary and sufficient for the development of tau pathology. Hyperphosphorylation of tau is found within dystrophic neurites surrounding β-amyloid deposits in AD mouse models but the pathological conversion of tau is absent. Likewise, expression of a human tau repeat domain in mice is insufficient to drive the pathological conversion of tau. Here we developed an Aβ-amyloidosis mouse model that expresses the human tau repeat domain and show that in these mice, the neuritic plaque facilitates the pathological conversion of wild-type tau. We show that this tau fragment seeds the neuritic plaque-dependent pathological conversion of wild-type tau that spreads from the cortex and hippocampus to the brain stem. These results establish that in addition to the neuritic plaque, a second determinant is required to drive the conversion of wild-type tau.

## Abstract (web summary)

Alzheimer's disease (AD) is pathologically characterized by the accumulation of neuritic plaques and neurofibrillary tangles but it is not known whether the neuritic plaque is necessary to drive the conversion of wild-type tau. Here the authors developed a mouse model in which wild-type tau is converted into pathological tau in a neuritic plaque-dependent manner.

## Graphical abstract

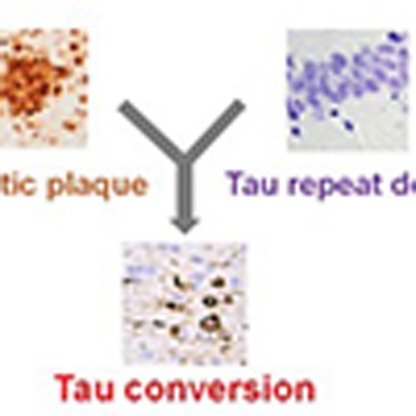

Alzheimer's disease (AD), the most common cause of dementia, is pathologically characterized by the accumulation of neuritic plaques and neurofibrillary tangles (NFT), with widespread gliosis, loss of synapses and degeneration of neurons^1^,^2^. The core of neuritic plaques consists of extracellular deposits of Aβ^3^ surrounded by astrocytes and microglia. The NFT is comprised of intracellular paired helical filaments derived from hyperphosphorylated microtubule binding protein, tau^4^,^5^. Genetic studies of early-onset familial AD (fAD), which accounts for 5–10% of cases of AD^6^,^7^, have fuelled the notion that abnormal accumulation of Aβ in the brain would trigger the aggregation of tau leading to neurodegeneration and dementia^2^,^8^. While previous studies showed that accumulation of Aβ could facilitate tau aggregation in FTDP-17 linked mutant Tau mice^9^,^10^,^11^,^12^,^13^, no such evidence have been demonstrated using a non-mutant tau model. Furthermore, it is not known whether the neuritic plaque is necessary and sufficient to drive the conversion of wild-type tau. In contrast to mutations in *APP* and *Presenilins* linked to fAD^14^,^15^, genetic risk alleles, including *ApoE*^16^,^17^,^18^,^19^ and *Trem2* (refs 20, 21), are associated with late-onset AD (LOAD), which constitutes ∼90% of AD cases. The notion that accumulation of Aβ is not sufficient to drive cognitive decline in LOAD is strongly supported by the following: a high Aβ burden is found in brains of some elderly in their eighties or nineties who remain cognitively normal^22^,^23^,^24^; and there is no correlation of Aβ plaques with cognition in LOAD^25^. In contrast, the aggregation of tau correlates with cognitive decline and is thought to drive neurodegeneration in AD^26^,^27^. This raises the intriguing notion that in addition to the Aβ burden, other risk factors help provoke the pathological conversion of tau to drive neuron loss and cognitive decline in LOAD. Resolution of this question will impact our view regarding disease mechanisms and identification of targets, and validate and test novel therapeutic strategies for LOAD.

Animal model systems have been instrumental in clarifying the molecular mechanisms and testing therapeutic strategies for AD^28^,^29^,^30^,^31^,^32^,^33^. Notwithstanding advances made using current rodent models^10^,^34^,^35^,^36^,^37^,^38^,^39^,^40^, a major limitation in the field is the lack of a rodent model that faithfully reproduce the tau pathology seen in humans with AD. Because no mutation in the *tau* gene has been identified in fAD or LOAD patients and expression of human wild-type tau failed to elicit tau pathology^41^, current mouse models of tau pathology are primarily based on transgenes expressing tau mutants linked to FTDP-17 (refs 9, 10, 11, 35, 36, 37, 42). However, this raises major concerns regarding their relevance to the disease context. It is well recognized that the tau pathology occurring in AD invariably depends on the conversion of the wild-type tau, a prion strain that is distinct from other human tauopathies such as that associated with FTDP-17 (ref. 43). Moreover, the tau pathology occurring in these FTDP-17-linked tau models is sufficient to drive cell death independent of Aβ plaques^44^, supporting the view that these mice may be good models of FTDP-17, but not of AD. To address this critical unmet need, we developed a mouse model in which wild-type tau is converted into pathological tau aggregates and NFT that are propagated through neuronal circuits to drive neuron loss in a neuritic plaque-dependent manner. Our results offer novel mechanistic insight for the pathogenesis of AD.

## Results

### Neuritic plaque is not sufficient for the conversion of tau

**Figure 1:**
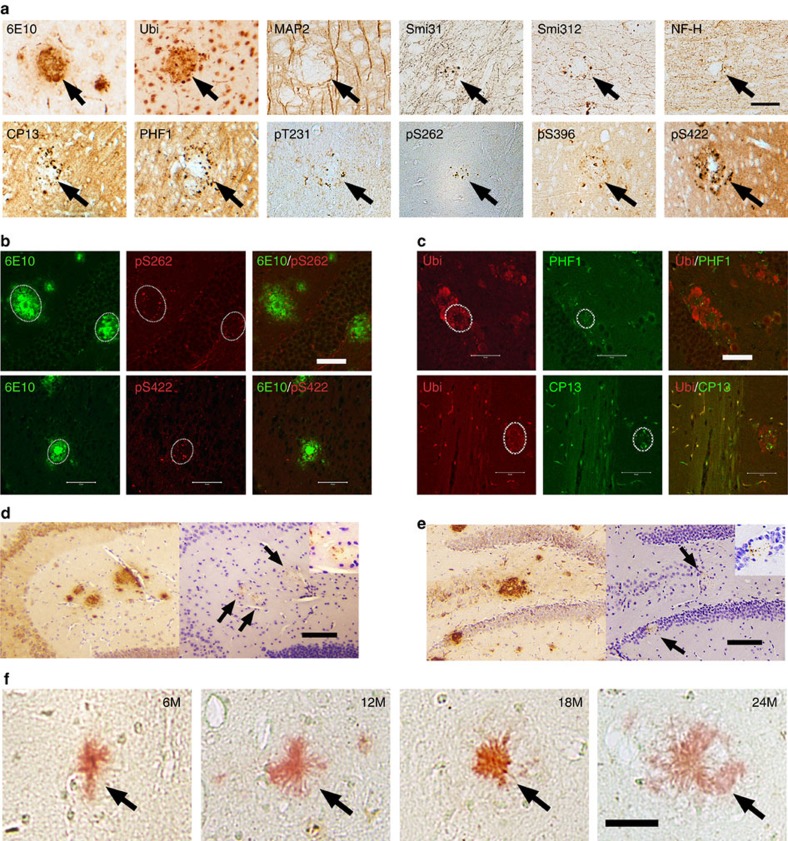
Neuritic plaques stimulate the phosphorylation of tau. (**a**) Brain sections of *APP*^*swe*^*;PS1ΔE9* mice (*n*=7) were detected by antibodies specific to Aβ (6E10), ubiquitin, Microtubule-associated protein 2 (Map2), neurofilament (Smi31, Smi312, and NF-H), and antibodies specific to phosphorylated tau: CP13 and PHF-1, Tau pT231, Tau pS262, Tau pS396, and Tau-pS422. Note the accumulation of phosphorylated tau surrounding the neuritic plaques. Scale bar, 50 μm. (**b**) Confocal microscopic analysis of Aβ and tau in cortex of *APP*^*swe*^*;PS1ΔE9* mice (*n*=6). Brain sections co-stained with antiserums specific to: Aβ (6E10) and tau (pS262) (upper panel); or Aβ (6E10) and tau (pS422) (lower panel). Scale bar, 50 μm. (**c**) Confocal microscopic analysis of Ubiquitin and tau in cortex of *APP;PS1ΔE9* mice (*n*=6). Brain sections co-stained with antiserums specific to: Ubiquitin and tau (CP13) (upper panel); or Ubiquitin and PHF-1 (lower panel). Scale bar, 50 μm. (**d**) Accumulation of phosphorylated tau in dystrophic neurites surrounding the central Aβ core in 6-month-old *APP*^*swe*^*;PS1ΔE9* mice (*n*=9) as detected by antibodies specific to Aβ (6E10) and phosphorylated tau (pS422). Scale bar, 100 μm. (**e**) Accumulation of phosphorylated tau in dystrophic neurites surrounding the central Aβ core in 12-month-old *APP*^*swe*^*;PS1ΔE9* mice (*n*=11) as detected by antibodies specific to Aβ (6E10) and phosphorylated tau (pS422). Scale bar, 100 μm. (**f**) No Gallyas positive tau tangle was detected around the Aβ core of neuritic plaques (Congo red, arrows) in *APP*^*swe*^*;PS1ΔE9* mice (*n*=9), even up to 24 months. Scale bar, 25 μm.

Although it is well recognized that endogenous tau can be hyperphosphorylated in mouse models of β-amyloidosis^40^, whether the neuritic plaque is necessary and sufficient to facilitate the pathological conversion of wild-type tau is not known. To address this issue, we examined first the biochemical alterations of endogenous tau occurring in *APP*^*swe*^*;PS1ΔE9* mice (a well-established mouse model of β-amyloidosis) during aging. Correlated with the presence of neuritic plaques was hyperphosphorylation of tau occurring within dystrophic neurites (Fig. 1a). The hyperphosphorylated tau was seen surrounding the central Aβ core but never co-localized with it (Fig. 1b); while closely associated, there is no direct physical interaction between the central Aβ core and the hyperphosphorylated tau. Moreover, the hyperphosphorylated tau was localized adjacent to ubiquitinated proteins within dystrophic neurites (Fig. 1c). Because tau was not hyperphosphorylated before the formation of neuritic plaques and its biochemical alteration persisted during aging (Fig. 1d,e), we infer that the neuritic plaque serves as the crucible that facilitates the hyperphosphorylation of tau. However, this persistent neuritic plaque-dependent biochemical alteration of tau failed to convert to tangle-like aggregates even in aged *APP*^*swe*^*;PS1ΔE9* mice that were beyond 24 months as judged by Gallyas preparation (Fig. 1f), a silver stain method for detecting conformational changes in tau. While the neuritic plaque is capable of stimulating the phosphorylation of tau, it is not sufficient to alter the conformation of tau to form tangles and drive the loss of neurons.

### Tau repeat domain is insufficient for the conversion of tau

**Figure 2:**
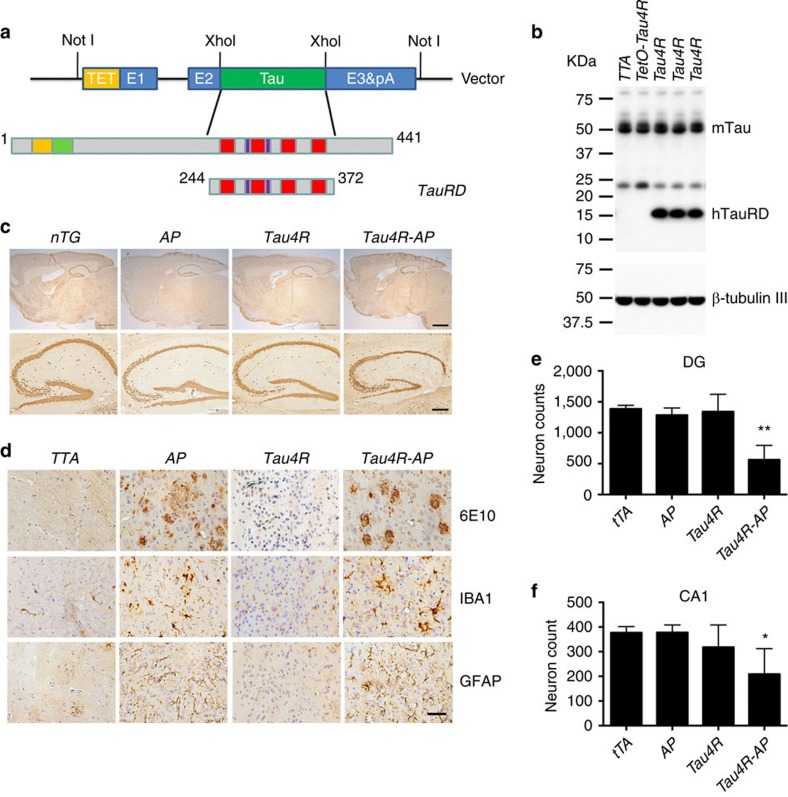
Neuritic plaque is required for the pathological conversion of tau. (**a**) Diagram depicting the expression construct of four-repeat domain of tau (*TauRD*). The top diagram represents the longest isoform of the human tau40 (441 residues). The bottom diagram shows the construct containing four-repeat domain of tau with (*TauRD*). (**b**) Protein blot using 77G7 antibody that recognized the repeat domain of tau showed the presence of exogenous (∼16 kDa) TauRD and endogenous tau protein from brain lysates of tau transgenic (*Tau4R*) mice (*n*=5). The expression level of tau in *Tau4R* mice is similar to that of non-transgenic mice. (**c**) Immunohistochemical analysis of brains of 20-month-old *nTG* (*n*=5)*, TTA* (*n*=4)*, Tau4R* (*n*=6) and *Tau4R-AP* mice (*n*=5) using antiserum specific to NeuN to detect neurons; sagittal sections of brains (upper panels; scale bar, 1,000 μm) and hippocampi (lower panels; scale bar, 200 μm). Note forebrain atrophy and reduction of neurons in the cortical and hippocampal area of *Tau4R-AP* mice. (**d**) Immunohistochemical analysis using antiserum specific to Aβ (6E10), microglia (IBA1) and reactive astrocytes (GFAP). Scale bar, 50 μm. Immunohistochemial analysis of brains of *nTG* (*n*=5)*, TTA* (*n*=4)*, Tau4R* (*n*=6) and *Tau4R-AP* mice (*n*=5) using antiserum specific to Aβ (6E10), microglial (IBA1) and reactive astrocytes (GFAP). Note increase of microglial and hypertrophic GFAP positive astrocytes in cortex of 20-month-old *Tau4R-AP* mice. Scale bar, 50 μm. (**e**) Neuronal cell count of dentate gyrus region from 18 months old *TTA* (*n*=5), *AP* (*n*=5), *Tau4R* (*n*=5)*, Tau4R-AP* (*n*=5) mice using ImageJ analysis. (one-way analysis of variance (ANOVA), **P=0.0002) (**f**) Neuronal cell count of CA1 regions from 20 months old *TTA* (*n*=4), *AP* (*n*=6), *Tau4R* (*n*=6)*, Tau4R-AP* (*n*=5) mice using ImageJ analysis. (one-way ANOVA, *P=0.0134).

**Figure 3:**
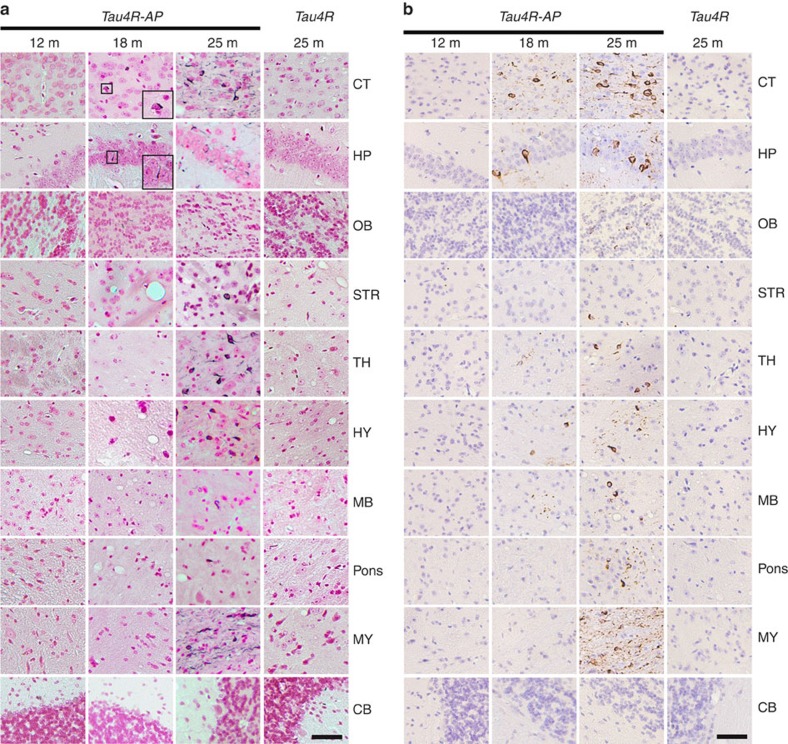
Age-dependent spreading of endogenous tau aggregates in brain regions of *Tau4R-AP* mice. (**a**) Gallyas-Braak silver staining of tau tangles in brain sections of *Tau4R-AP* mice at 12 (*n*=3), 18 (*n*=4), and 25 months of age (*n*=6). The sections were counterstained with fast red. The right panels are *Tau4R* mice at 25 months of age (*n*=6). The brain regions are cerebral cortex (CT); hippocampus (HP); olfactory bulb (OB); striatum (STR); thalamus (TH); hypothalamus (HY); midbrain (MB); Pons; Medulla (MY), and cerebellum (CB). Tau tangles could be detected in *Tau4R-AP*, but not *Tau4R*, mice at 18 months of ages, and the accumulation of tangles were dramatically increased with aging and spread to other brain regions (25 months of age, third column), except in cerebellum (bottom panel). Scale bar, 50 μm. (**b**) Immunohistochemical analysis showed age-dependent spreading of endogenous tau tangle in *Tau4R-AP* mice (*n*=13) using antibodies specific to endogenous phosphorylated S422 of tau (pS422). The brain regions are cerebral cortex (CT); hippocampus (HP); olfactory bulb (OB); striatum (STR); thalamus (TH); hypothalamus (HY); midbrain (MB); Pons; Medulla (MY), and cerebellum (CB). While no signal was detected in *Tau4R* mice (*n*=6) even at 25 months of age, tau tangles first appeared in cortical and hippocampal region (18 months of age, second column) of *Tau4R-AP* mice and spread to other brain region with aging (25 months of age, third column), except in cerebellum (bottom panel). Scale bar, 50 μm.

The insufficiency of the neuritic plaque to drive the pathological conversion of tau suggested that a second-risk determinant may be required. That accumulating evidence indicated that other types of tau modification, such as fragmentation^45^ or acetylation^46^,^47^, facilitate its aggregation encouraged us to determine whether a tau repeat domain could facilitate the pathological conversion of wild-type tau. We used the *moPrP-tet* vector^48^ to express a human four-repeat domain of tau (Q244-E372, TauRD) in the nervous system of mice under the control of the tetracycline transactivator (tTA) (Fig. 2a). From multiple lines, we selected for subsequent studies one *Tau4R* line, which accumulated the human tau fragment to ∼1.1-fold of endogenous tau (Fig. 2b, Supplementary Fig. 1). No difference can be detected in the overall structure and weight of brains of *Tau4R* mice even at 25 months of age (Fig. 2c, third column). Histological analysis confirmed no evidence of activation of astrocytes/microglia or neuronal loss in *Tau4R* mice (Fig. 2d, third column). We also failed to observe hyperphosphorylation of tau or formation of tau tangle (Fig. 3a,b, forth column), indicating that the tau repeat domain alone is insufficient to drive the pathological conversion of wild-type tau.

### Tau fragment seeds neuritic plaque-dependent tau conversion

Although the human tau repeat domain was not sufficient to alter the conformation of endogenous tau, we asked whether such fragmentation of tau could serve as the second-risk determinant in the presence of neuritic plaques to drive tau pathology. To address this question, we crossbred *Tau4R* mice with *APP*^*swe*^*;PS1ΔE9* mice^49^ to generate compound *Tau4R;APP*^*swe*^*;PS1ΔE9* (*Tau4R-AP*) mice. *Tau4R-AP* mice exhibited marked forebrain atrophy (Fig. 2c) with significant neuronal loss in dentate gyrus or CA1 region of the hippocampus, respectively, at 18 (Fig. 2e) or 20 (Fig. 2f) month of age; the loss of neurons in the cortex and hippocampus is associated with the accumulation of phosphorylated tau and NFT (Fig. 3a,b). Taken together with our finding that the neuritic plaque is required for the phosphorylation of tau, these results indicate that the fragmentation of tau could seed the neuritic plaque-dependent pathological conversion of wild-type tau.

To access the evolution of tau tangles in *Tau4R-AP* mice, we performed a time series focusing on the pattern of distribution of mouse tau aggregates. Whereas tau aggregates were undetectable before 1 year of age, they were first detected in hippocampus and cortical regions by 15 months of age and significantly increased by 18 months of age (Fig. 3a). By 2 years, tau tangles were readily observed throughout the whole brain except the cerebellum (Fig. 3a), suggesting that the tau tangle initiated from the frontal area is able to spread to the rest of the brain through neuronal circuits. Because the pattern and intensity of Gallyas stain paralleled (Fig. 3a) those of the immunocytochemical staining of misfolded mouse tau (Fig. 3b), these tau tangles would be principally derived from the endogenous tau, especially in the brain stem and midbrain where the human tau fragment was not expressed. These findings indicate that while neither the neuritic plaque nor the human tau repeat domain is sufficient to drive the pathological conversion of wild-type tau, they both are required for the seeding and aggregation of the tau repeat domain, which facilitate the pathological conversion of mouse wild-type tau that spread from the cortex and hippocampus to the brain stem.

### Neuritic plaque is necessary for the conversion of tau

**Figure 4:**
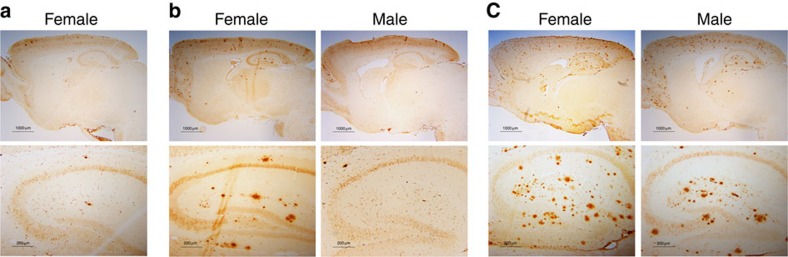
Gender difference in onset of neuritic plaques in *APP*^*swe*^*;PS1ΔE9* mice. (**a**) Brain sections of 4-month-old female (*n*=5) *APP*^*swe*^*;PS1ΔE9* mice were detected by antibodies specific to Aβ (6E10). Only a few can be detected in the female *APP*^*swe*^*;PS1ΔE9* mice at 4 months of age. (**b**) Brain sections of 6-month-old female (*n*=6) and male (*n*=4) *APP*^*swe*^*;PS1ΔE9* mice were detected by antibodies specific to Aβ (6E10). Wide spread neuritic plaques are observed in female *APP*^*swe*^*;PS1ΔE9* mice, while only a few can be detected in the male *APP*^*swe*^*;PS1ΔE9* mice at 6 months of age. (**c**) Brain sections of 12-month-old female (*n*=9) and male (*n*=7) *APP*^*swe*^*;PS1ΔE9* mice were detected by antibodies specific to Aβ (6E10). Wide spread neuritic plaques are observed in both male and female *APP*^*swe*^*;PS1ΔE9* mice at this age.

**Figure 5:**
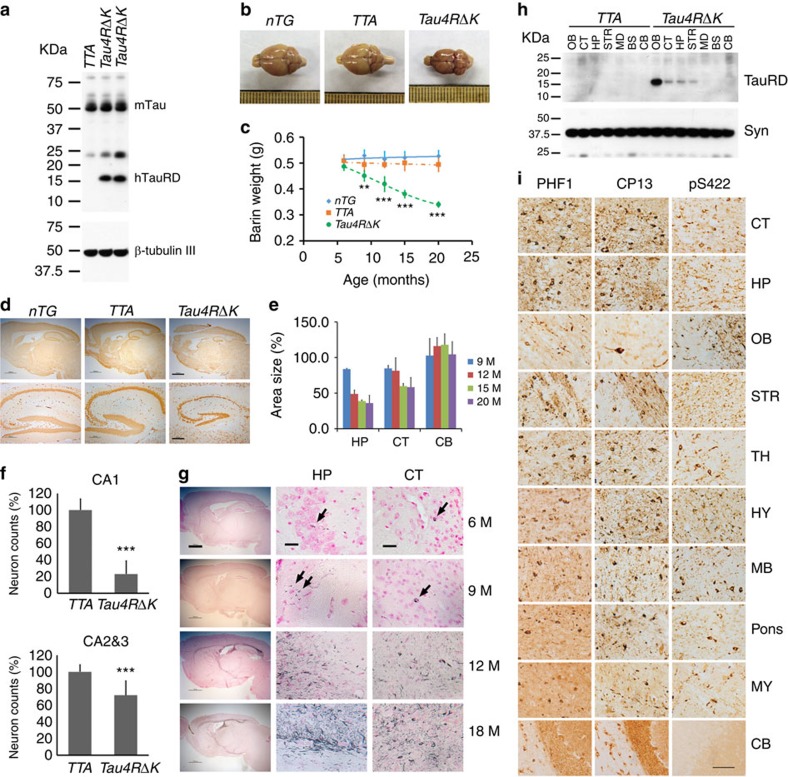
Pathological conversion of tau induced by a mutant tau repeat domain. (**a**) Protein blot using 77G7 antibody to detect exogenous (∼16 kDa) TauRDΔK and endogenous tau protein from brain lysates of mutant tau transgenic (*Tau4RΔK*) mice (*n*=9). (**b**) Representative brains of *nTG, TTA and Tau4RΔK* mice at 20 months of age. Note marked forebrain atrophy in *Tau4RΔK* mice (*n*=7). (**c**) The plot of brain weight of *nTG* (*n*=29)*, TTA* (*n*=27) and *Tau4RΔK* (*n*=24) mice at different of ages. The brain weight of *Tau4RΔK* mice is progressively reduced with aging. (one-way analysis of variance (ANOVA), 9 M **P=0.02; 12 M ***P=0.0001; 15 M ***P=0.0003; 20 M ***P=0.0006). (**d**) Immunohistochemical analysis using antiserum specific to NeuN: sagittal sections (upper panels; scale bar, 1,000 μm) and hippocampi (lower panels; scale bar, 200 μm) of 18-month-old *Tau4RΔK* (*n*=8) and control mice (*n*=15). (**e**) Sizes of cortical (CT), hippocampal (HP) and cerebellum (CB) regions in brains of *Tau4RΔK* (*n*=24) at various ages. Note reduction of hippocampus and cortical region, but not cerebellum in *Tau4RΔK* mice. (**f**) Neuronal cell count of CA1 (*T*-Test, ***P=4.45E^−09^) and CA2&3 (*T*-Test, ***P=0.00033) region from 12 months old *TTA* (*n*=7)*, Tau4RΔK* (*n*=7) mice using ImageJ analysis. (**g**) Gallyas-Braak silver staining of brain sections of *Tau4RΔK* mice at various ages. The sections were counterstained with fast red. The left panel is overview image of the sagittal section of brains (Scale bar, 1,000 μm). The middle and right panels are hippocampal (HP) and cortical regions (CT). (Scale bar, 25 μm). Tau tangle could first be detected at 6 months of age, and the accumulation of tangles were dramatically increased while aging. (**h**) Total protein was extracted from different brain regions: olfactory bulb (OB), cortex (CT), hippocampus (HP), striatum (STR), midbrain (MB), brain stem (BS), cerebellum (CE) of 9 months old *TTA* (*n*=3) and *Tau4RΔK* (*n*=3) mice. Human tau fragment (∼16 kDa) detected using anti-human tau polyclonal antiserum KJ9A was only seen in frontal region, but not in MB, BS or CE. (**i**) Immunohistochemial analysis of brains of 20 months old *Tau4RΔK* mice (*n*=5) by antibodies specific to phosphorylated endogenous tau: PHF-1 (left panel), CP13 (middle panel), and tau-pS422 (right panel), respectively. Scale bar, 50 μm.

To test directly whether the neuritic plaque is required for the pathological conversion of wild-type tau, we took advantage of the gender difference in onset of neuritic plaques occurring in *APP*^*swe*^*;PS1ΔE9* mice; onset for females or males was, respectively, at 4 or 6 months of age (Fig. 4). We would anticipate that if formation of the neuritic plaque precedes tau pathology, the pathological conversion of wild-type tau should be accelerated; otherwise, no such effect would be expected. For such a test, a mouse model in which the onset of tau pathology occurred after the onset of neuritic plaque in female *APP*^*swe*^*;PS1ΔE9* mice, but similar to that of males, would be necessary. We therefore generated mice expressing the four-repeat domain of tau (Q244-E372) harbouring a pro-aggregation ΔK280 mutation (*Tau4RΔK*), previously shown to induce tau pathology in mice^50^. From multiple lines, we selected one line of *Tau4RΔK*, in which the exogenous tau fragments accumulated to 0.7-fold of endogenous tau (Fig. 5a, Supplementary Fig. 1). Brains of *Tau4RΔK* mice showed a progressive and marked atrophy of their forebrains (Fig. 5b) accompanied over time by a decline in the weight of brains (Fig. 5c). Histopathological analysis confirmed a marked decrease in size of the hippocampus (Fig. 5d,e) and cortex (Fig. 5e) and a marked reduction in the number of neurons by 12 months of age (Fig. 5f). Tau tangles first appeared at 6 months of age (Fig. 5g, upper panel), which were dramatically increased by 12 months (Fig. 5g) and except for the cerebellum, spread throughout the brain (Fig. 5g, lower panel) by 18 months. Moreover, aggregations derived from the endogenous wild-type tau exhibited a similar pattern (Fig. 5i), including the midbrain and brain stem. However, the presence of the human tau fragment was restricted to frontal area of the brain (Fig. 5h, Supplementary Fig. 2), suggesting that the exogenous tau fragment serves as a seed to convert the wild-type tau to tangles, which spread in a prion mechanism to other regions of the brain.

**Figure 6:**
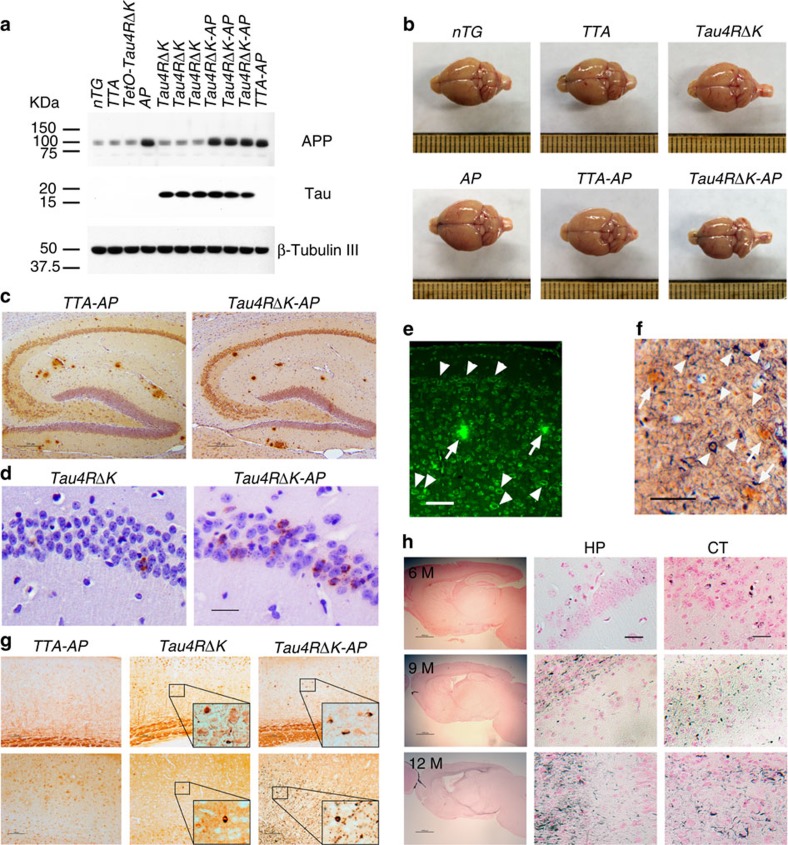
Neuritic plaque-dependent acceleration of pathological conversion of tau induced by a mutant tau repeat domain. (**a**) Protein blot analysis of tau and APP in brain lysates of 3 month-old female *TTA* (*n*=6)*, tetO-tauRDΔK* (*n*=6)*, AP* (*n*=3), *TTA-AP* (*n*=7), *Tau4RΔK* (*n*=5)*, Tau4RΔK-AP* (*n*=4) and *nTG* (*n*=7) mice using antisera K9JA and CT15. (**b**) Representative brains of 9 month-old female *TTA, AP*, *TTA-AP*, *Tau4RΔK, Tau4RΔK-AP* and *nTG* mice. Note marked forebrain atrophy in *Tau4RΔK* mice. (**c**) Immunostaining of brain sections of 6-month-old female *TTA-AP* (*n*=7) and *Tau4RΔK-AP* mice (*n*=5) with antibodies 6E10 specific to human Aβ. Scale bars, 200 μm. (**d**) Immunostaining of 6 month-old female brain sections of *Tau4RΔK* (*n*=4), and *Tau4RΔK-AP* mice (*n*=4) using antiserum tau-pS422. Scale bar, 50 μm. (**e**) Aβ plaques (arrows) and abundance of tau aggregates (arrowheads) were observed in *Tau4RΔK-AP* mice (*n*=5) at 9 months of age by Thioflavin-T staining. Scale bar, 50 μm. (**f**) Silver staining to detect Aβ plaques (arrows) and tau tangles (arrowheads) in brain of *Tau4RΔK-AP* mice (*n*=5). Scale bar, 50 μm. (**g**) Immunostaining of brain sections of 9 month-old female *Tau4RΔK* (*n*=5), and *Tau4RΔK-AP* mice (*n*=4) using tau-pS422. Scale bar, 100 μm. (**h**) Gallyas-Braak silver staining of brain sections of female *Tau4RΔK-AP* mice at 6 (top panel, *n*=4), 9 (middle panel, *n*=6) and 12 (bottom panel, *n*=3) months of age (counterstained with fast red). The left panel: sagittal section (Scale bar, 1,000 μm); middle and right panel are hippocampal (HP) and cortical regions (CT), respectively (Scale bar, 25 μm).

**Figure 7:**
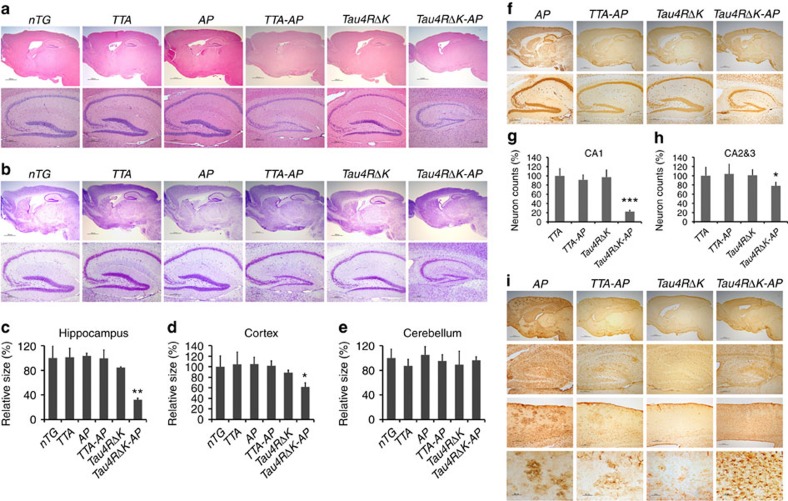
Acceleration of neuronal loss, astrocytosis and forebrain atrophy in *Tau4RΔK-AP* mice. (**a**) Hematoxylin and eosin (H&E) staining of sagittal sections of brains (upper panels; scale bar, 1,000 μm) and hippocampi (lower panels; scale bar, 200 μm) of 9-month-old female *TTA, APP*^*swe*^*;PS1ΔE9(AP)*, *TTA;APP*^*swe*^*;PS1ΔE9* (*TTA-AP*), *Tau4RΔK, Tau4RΔK;APP*^*swe*^*;PS1ΔE9* (*Tau4RΔK-AP*) and non-transgenic *(nTG)* mice. Note forebrain atrophy and reduction of neurons in the cortical and hippocampal area of *Tau4RΔK-AP* mice. (**b**) Cresyl violet (CV) staining of sagittal sections of brains (upper panels; scale bar, 1,000 μm) and hippocampi (lower panels; scale bar, 200 μm) of 9-month-old female *TTA, APP*^*swe*^*;PS1ΔE9(AP)*, *TTA;APP*^*swe*^*;PS1ΔE9* (*TTA-AP*), *Tau4RΔK, Tau4RΔK;APP*^*swe*^*;PS1ΔE9* (*Tau4RΔK-AP*) and non-transgenic *(nTG)* mice. Note reduction of neurons in *Tau4RΔK-AP* mice. (**c**–**e**) Sizes of hippocampal (**c**), cortical (**d**) and cerebellum (**e**) regions in brains of 9 months old *TTA* (*n*=6)*, AP* (*n*=7), *TTA-AP* (*n*=6), *Tau4RΔK* (*n*=5)*, Tau4RΔK-AP* (*n*=4) and *nTG* (*n*=15). Note reduction of hippocampus (one-way analysis of variance (ANOVA), **P=0.00052) and cortical region (one-way ANOVA, *P=0.014), but not cerebellum in *Tau4RΔK-AP* mice (**f**) Immunohistochemical analysis of brains of 9 months old *APP*^*swe*^*;PS1ΔE9*, *TTA-AP*, *Tau4RΔK* and *Tau4RΔK-AP* mice using antiserum specific to NeuN to detect neurons; sagittal sections of brains (upper panels; scale bar, 1,000 μm) and hippocampi (lower panels; scale bar, 200 μm). Note forebrain atrophy and reduction of neurons in the cortical and hippocampal area of *Tau4RΔK-AP* mice. (**g**) Neuronal cell count of CA1 region from 9 months old *TTA* (*n*=5), *TTA-AP* (*n*=6), *Tau4RΔK* (*n*=5)*, Tau4RΔK-AP* (*n*=5) mice using ImageJ analysis. Note ∼80% reduction of neurons in *Tau4RΔK-AP* mice. (***, One-way ANOVA, *P*=2.04E-06) (**h**) Neuronal cell count of CA2 and CA3 regions from 9 months old mice using ImageJ analysis. Significant reduction (∼20%) of neurons are observed of *Tau4RΔK-AP* mice as compared with those of control mice. (one-way ANOVA, *P=0.0133) (**i**) Immunohistochemial analysis of brains of *APP*^*swe*^*;PS1ΔE99*, *TTA-AP, Tau4RΔK* and *Tau4RΔK-AP* mice at 9 months of age using antiserum against GFAP: sagittal section (top panel, scale bar, 1,000 μm); hippocampi (second panel, scale bar, 200 μm); cortex (third panel, scale bar, 200 μm); higher power views of cortex (fourth panel, scale bar, 50 μm). Note hypertrophic GFAP positive astrocytes in cortex of *Tau4RΔK-AP* mice.

**Figure 8:**
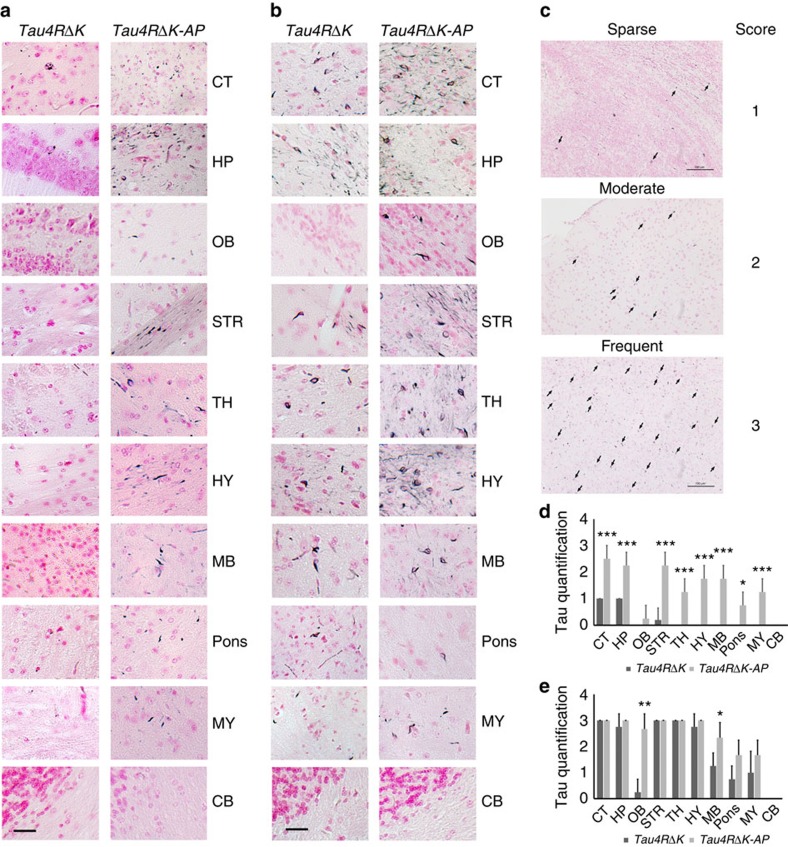
Acceleration of tau pathology in female *Tau4RΔK-AP* mice. (**a**) Gallyas-Braak silver staining of brain sections of female *Tau4RΔK* (*n*=5) and *Tau4RΔK-AP* mice (*n*=4) at 9 months of age (counterstained with fast red). Cerebral cortex (CT); hippocampus (HP); olfactory bulb (OB); striatum (STR); thalamus (TH); hypothalamus (HY); midbrain (MB); Pons; Medulla (MY), and cerebellum (CB) were shown, respectively. While only sparse tau tangle was detected in frontal area of the *Tau4RΔK* mice, wide spread tau tangle and thread were observed in *Tau4RΔK-AP* mice, including brain stem. (Scale bar, 25 μm). (**b**) Gallyas-Braak silver staining of brain sections of female *Tau4RΔK* (*n*=4) and *Tau4RΔK-AP* mice (*n*=3) at 12 months of age (counterstained with fast red). Cerebral cortex (CT); hippocampus (HP); olfactory bulb (OB); striatum (STR); thalamus (TH); hypothalamus (HY); midbrain (MB); Pons; Medulla (MY), and cerebellum (CB) were shown, respectively. Note more tau tangles were detected in different brain regions of *Tau4RΔK-AP* mice as compare with that of *Tau4RΔK* mice. (Scale bar, 25 μm). (**c**) Semiquantificative score of tau tangle frequency in low power (200X) microscope field. Score was indicated as: 0, no tangle; 1, 1–5 tangles per field; 2, 6–15 tangles per field; and 3, >15 tangles per field. (Scale bar, 100 μm) (**d**) Score of tau tangle frequency in different brain regions of 9 months old female *Tau4RΔK* (*n*=5) and *Tau4RΔK-AP* (*n*=4) mice. Cerebral cortex (CT; *T*-Test, ***P=0.0006); hippocampus (HP; *T*-Test, ***P=0.0007); olfactory bulb (OB); striatum (STR; *T*-Test, ***P=0.0003); thalamus (TH; *T*-Test, ***P=0.0007); hypothalamus (HY; *T*-Test, ***P=0.0001); midbrain (MB; *T*-Test, ***P=0.0001); Pons(*T*-Test, *P=0.01); Medulla (MY; *T*-Test, ***P=0.0007), and cerebellum (CB) were shown. While tau tangles were only detected in frontal region in *Tau4RΔK* mice, widespread tau tangles were observed in whole brain of *Tau4RΔK-AP* mice, including brain stem. (**e**) Score of tau tangle frequency in different brain regions of 12 months old female *Tau4RΔK* (*n*=4) and *Tau4RΔK-AP* (*n*=3) mice. CT; HP; OB (*T*-Test, **P=0.002); STR; TH; HY; MB, (*T*-Test, *P=0.045); Pons; MY, and CB region were shown. Note more frequent tau tangles detected in different regions of *Tau4RΔK-AP* mice compared with that of *Tau4RΔK* mice, especially in olfactory bulb and brain stem.

Because the onset of tau pathology in *Tau4RΔK* mice occurs after the onset of neuritic plaque formation in the female *APP*^*swe*^*;PS1ΔE9* mice (∼4 months of age, Fig. 4a), but earlier or at the same time as that of males (∼6 months of age, Fig. 4b), we took advantage of this sex difference to determine whether neuritic plaques could accelerate the conversion of wild-type tau to tangles. We crossbred *Tau4RΔK* mice with *APP*^*swe*^*;PS1ΔE9* mice^49^ to generate compound *Tau4RΔK;APP*^*swe*^*;PS1ΔE9* (*Tau4RΔK-AP*) mice. The expression level of exogenous tau in *Tau4RΔK-AP* mice was comparable to that of the *Tau4RΔK* mice (Fig. 6a, Supplementary Fig. 3), indicating that the Aβ burden did not influence the expression of tau. Compared with female *APP*^*swe*^*;PS1ΔE9* mice, the amount of Aβ deposit in 6-month-old female *Tau4RΔK-AP* mice remained unchanged (Fig. 6c). As expected, we observed marked increase in accumulation of phosphorylated tau in female *Tau4RΔK-AP* mice by 6 months of age (Fig. 6d). By 9 months of age, female *Tau4RΔK-AP* mice exhibited marked reduction in brain size (Fig. 6b) accompanied by widespread Aβ and tau pathologies (Fig. 6e,f). Histological analysis confirmed accelerated atrophy of forebrains in 9-month-old female *Tau4RΔK-AP* mice(Fig. 7a,b); specifically, sizes of hippocampus and cortex were significantly reduced (Fig. 7c,d). As expected, the cerebellum (Fig. 7e) of these *Tau4RΔK-AP* mice remained normal as tau pathology was absent in this region. To determine the impact of neuritic plaques, we examined neuron loss in brains of 9 month-old female *Tau4RΔK-AP* mice (Fig. 7f). As expected, marked reduction in number of neurons was observed in the cortical and hippocampal area of 9-month-old female *Tau4RΔK-AP*, but not female *Tau4RΔK* mice (Fig. 7g,h). An 80% and 25% reduction, respectively, in number of neurons were observed in CA1 (Fig. 7g) as well as CA2 and CA3 regions (Fig. 7h). Moreover, the development of phosphorylated tau aggregates (Fig. 6g) and tau tangles (Fig. 6h) was greatly accelerated in female *Tau4RΔK-AP* mice. Using Gallyas silver staining, we observed as early as 9 months of age tau tangles that already spread to the brain stem of *Tau4RΔK-AP*, but not *Tau4RΔK*, mice (Fig. 8a,c,d); tau tangles in *Tau4RΔK* mice were seen only at a later time point and by 12 months of age, a greater tau burden was found throughout the brain of *Tau4RΔK-AP* mice (Fig. 8b,c,e). Hypertrophic GFAP positive astrocytes were observed in cortical regions of *Tau4RΔK-AP*, but not *Tau4RΔK*, mice (Fig. 7i), indicating that reactive astrogliosis is correlated with the loss of neurons. These results indicate that neuritic plaques accelerate the onset of tau aggregation.

**Figure 9:**
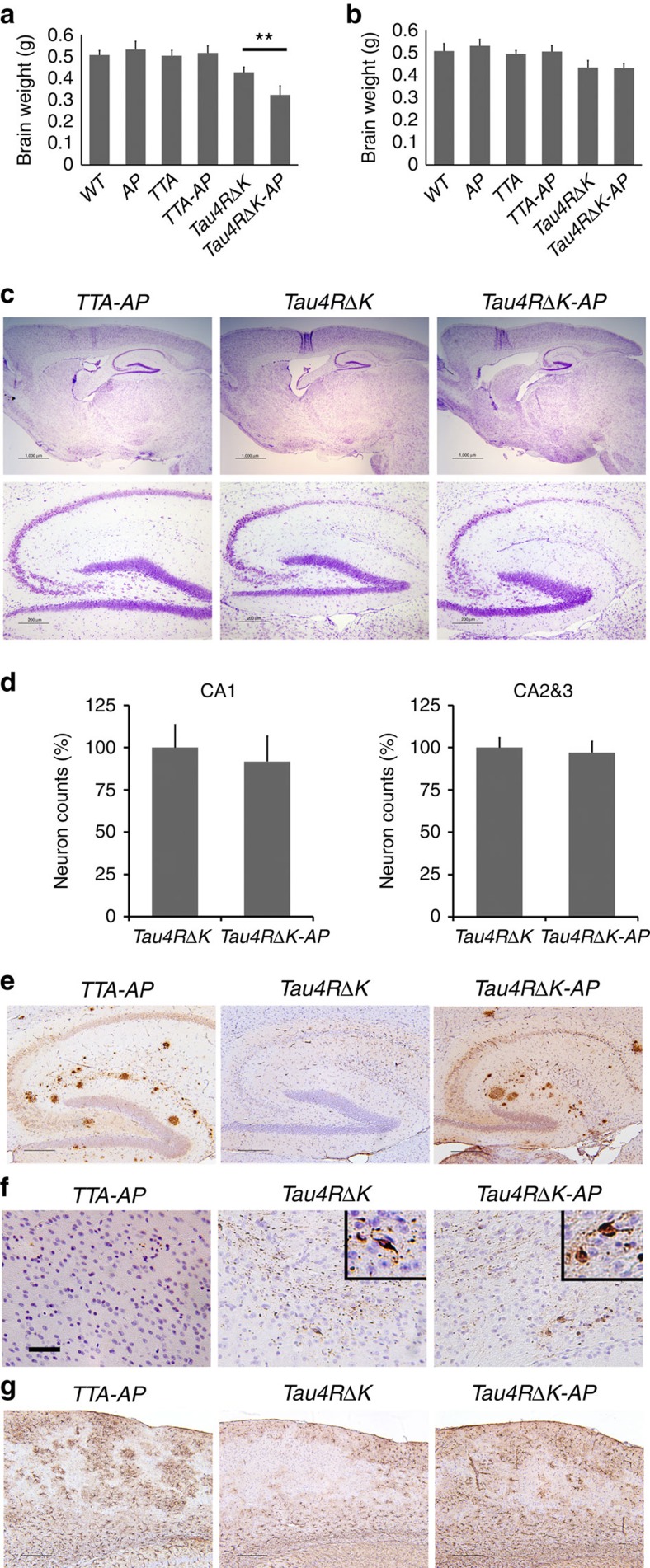
Acceleration of onset of tau pathology is dependent on the Neuritic plaque. (**a**) Brain weight of 12-month-old female *nTG* (*n*=11)*, AP* (*n*=9)*, TTA* (*n*=7)*, TTA-AP* (*n*=7), *Tau4RΔK* (*n*=5) and *Tau4RΔK-AP* (*n*=4) mice. The brain weight of *Tau4RΔK-AP* mice is 25% smaller than that of the *Tau4RΔK* mice at 12 month of age. (one-way analysis of variance, **P=0.002). (**b**) Brain weight of 12 month-old male *nTG* (*n*=11)*, AP* (*n*=6)*, TTA* (*n*=6)*, TTA-AP* (*n*=7), *Tau4RΔK* (*n*=5) and *Tau4RΔK-AP* (*n*=5) mice. No significant difference in brain weight is detected between the male *Tau4RΔK-AP* and *Tau4RΔK* mice at 12 month of age. (**c**) CV staining of brain sections of 11-month-old male *TTA-AP, Tau4RΔK* and *Tau4RΔK-AP* mice brains (upper panels; scale bar, 1,000 μm; and hippocampi, lower panels; scale bar, 200 μm). (**d**) Neuronal cell count of CA1, and CA2&3 region from 11 months old male *Tau4RΔK* (*n*=6) and *Tau4RΔK-AP* (*n*=4) mice. No significant difference was observed between the two mouse lines. (**e**) Immunostaining of brain sections of 11-month-old male *TTA-AP* (*n*=5)*, Tau4RΔK* (*n*=5) and *Tau4RΔK-AP* mice (*n*=4) with antibodies (6E10) specific to human Aβ. Scale bars, 200 μm. (**f**) Immunostaining of brain sections of 11 month-old male *TTA-AP* (*n*=5)*, Tau4RΔK* (*n*=5), and *Tau4RΔK-AP* (*n*=4) mice using tau-pS422. Scale bar, 100 μm. (**g**) Immunostaining of brain sections of 11-month-old *TTA-AP* (*n*=5)*, Tau4RΔK* (*n*=5) and *Tau4RΔK-AP* (*n*=4) mice with antibodies specific to GFAP. Scale bars, 200 μm.

However, tau pathology was not accelerated in male *Tau4RΔK-AP* mice as compared with that of *Tau4RΔK* mice. At 12 months of age, while marked acceleration of brain atrophy was observed in female *Tau4RΔK-AP* mice (Fig. 9a), no such accelerated atrophy of forebrains occurred in male *Tau4RΔK-AP* mice (Fig. 9b). Histological analysis showed similar level of brain atrophy (Fig. 9c) and neuronal loss in male *Tau4RΔK-AP* as compared with that of male *Tau4RΔK* mice (Fig. 9d). No significant changes in accumulation of Aβ plaques (Fig. 9e) or activated astrocytes (Fig. 9g) were observed in male *Tau4RΔK-AP* mice. The accumulation of tau aggregates was not significantly altered in male *Tau4RΔK-AP* mice as compared with that of male *Tau4RΔK* mice (Fig. 9f), indicating that neuritic plaques have little effect on the rate of tau aggregation after its onset. Taken together, these results establish that the neuritic plaque is required for the pathological conversion of wild-type tau.

## Discussion

Clarification of the key factors contributing to the pathological conversion of wild-type tau, a prion strain or conformation that is perhaps unique to AD^43^, could be instrumental for developing effective therapy for this devastating disease of the elderly. Our findings here establish that the neuritic plaque is required but not sufficient to drive the pathological conversion of wild-type tau. While previous effort primarily employed transgenes expressing tau mutants linked to FTDP-17 (refs 9, 10, 11, 35, 36, 37, 42) that are sufficient to aggregate independent of the neuritic plaque, we developed a mouse model expressing human tau repeat domain that is capable of inducing the pathological conversion of mouse wild-type tau only in the presence of the neuritic plaque. Our discovery provides a fundamental paradigm shift and a framework towards identification of risk alleles/factors that play essential roles in the pathological conversion of tau in AD.

**Figure 10:**
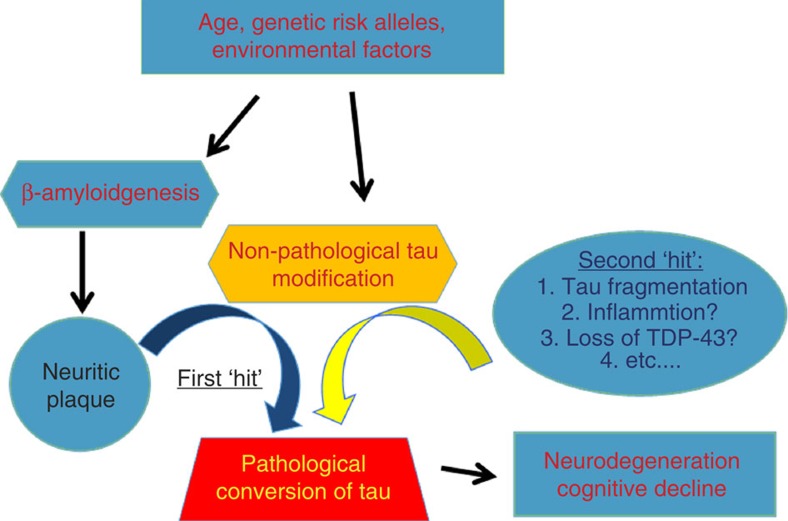
A multifactorial Model for LOAD. Diagram depicting that the neuritic plaque is required, but insufficient for the pathological conversion of tau. A second ‘hit' involving a variety of risk alleles/factors, would be necessary to facilitate the neuritic plaque-dependent pathological conversion of the wild-type tau in LOAD.

On the basis of our discovery, we propose here a model (Fig. 10) whereby the β-amyloid dependent formation of neuritic plaque during aging provides the molecular environment to facilitate the biochemical modification of tau within dystrophic neurites. While the neuritic plaque is necessary, it is not sufficient to convert the wild-type tau to a pathological conformation that drives neurodegeneration with ensuing cognitive decline and atrophy of the brain. In addition to the neuritic plaque, we envision a second-risk determinant (here we illustrate with the fragmentation of tau) would be required to drive the pathological conversion of wild-type tau. Thus, we propose that a combination of risk alleles/factors may facilitate the neuritic plaque-dependent pathological conversion of the wild-type tau in LOAD. Genetic studies has identified a series of risk alleles, in addition to *ApoE4* allele, that are associated with LOAD^51^,^52^,^53^,^54^. That subsets of individuals whose brains are littered with neuritic plaques remain cognitive normal^22^,^23^,^24^ can be explained by our ‘two-hit' hypothesis because they fail to harbour another risk allele that is required to drive the conversion of tau. Moreover, our model is also consistent with the long pre-symptomatic phase seen in LOAD^55^,^56^ during which time neuritic plaques accumulate because until the second-risk determinant is activated, tau remains unconverted. Likewise, our model predicts that cognitive decline in LOAD would be correlated with tau pathology rather than with neuritic plaques because once initiated, the tau aggregates are sufficient to propagate and spread throughout the brain via neuronal circuits and drive neuronal loss with ensuing cognitive decline. Thus, we believe that the pathogenesis of LOAD is a multifactorial problem initiated by the age-dependent formation of the neuritic plaque and activation of a variety of potential risk determinants to drive the pathological conversion of tau (Fig. 10).

From a therapeutic perspective, our model first predicts that therapies designed to prevent the conversion of tau would be most beneficial because the presence of neuritic plaques, in the absence of tau aggregates will not be sufficient to drive neuron loss. Our novel mouse model exhibiting age- and neuritic plaque-dependent tau pathology will now provide an invaluable platform for testing a variety of risk alleles/factors that may be relevant for the pathological conversion of tau. While a three-dimensional human neural cell culture system displaying Aβ and tau pathologies^57^ is a very useful tool for *in vitro* drug screening, our model exhibiting age-dependent development of neuritic plaques and tau pathologies that drives the progressive loss of neuron will be an essential *in vivo* tool for drug discovery programs designed to attenuate neurodegeneration for LOAD. Second, our model predicts that anti-amyloid therapies directed at early pre-symptomatic AD long before the pathological conversion of tau would slow disease by delaying the onset of tau pathology. In retrospect, perhaps it was not too surprising that outcomes of clinical trials with anti-amyloid agents in humans have been disappointing^58^,^59^,^60^,^61^ when delivered during a time when the conversion of tau has already initiated. Finally, our model predicts that a combinatorial therapy designed to prevent both neuritic plaque formation as well as pathological conversion of tau may provide optimal benefit for LOAD.

## Methods

### Generation of transgenic mice

To generate *tau* transgenic mice, DNA fragments encoding wild-type and ΔK280 mutant four-repeat domains of tau (TauRD, that is,Q244-E372) were subcloned into a previously characterized *moPrP-tetP* vector^48^, which expresses the gene consistently in neurons under the control of tTA proteins. The DNA was microinjected into C57BL/6 X SJL F2 mouse embryos (University of Michigan transgenic facility) to produce transgenic mice carrying wild-type (*TetO-TauRD)* or mutant (*TetO-TauRDΔK*) *Tau* fragment with regulatory element, *moPrP-tetP* promoter. Subsequently, these mice were crossbred with *CamKII-tTA* mice to bring the Tau transgene under the control of tet-off *CamKII* promoter^62^ (*Tau4R* and *Tau4RΔK* mice).

Doxycycline hydrochloride, dissolved in water to final concentration of 20 mg ml^−1^ and sterilized by filtration with 0.22 μm filter, was supplied in the drinking water to suppress the exogenous Tau expression during early development. During gestation and before weaning, the mice were raised in the presence of 50 μg ml^−1^ doxycycline hydrochloride.

We crossbred CamKII-tTA;TetO-TauRD or CamKII-tTA;TetO-TauRDΔK mice with APP^swe^;PS1ΔE9 mice^49^ to generate APP^swe^;PS1ΔE9;CamKII-tTA;TetO-TauRD (Tau4R-AP ) and APP^swe^;PS1ΔE9;CamKII-tTA;TetO-TauRDΔK (Tau4RΔK-AP) mice that develop both Tau pathology and Aβ amyloidosis; Tau4R or Tau4RΔK, CamKII-tTA (TTA), APP^swe^;PS1ΔE9;CamKII-tTA (TTA-AP), APP^swe^;PS1ΔE9 (AP) and non-transgenic littermates were used as controls. The brains of mice at various ages were collected, weighed and processed for biochemical, histological and immunohistochemical analysis.

All mice were maintained under 12L:12D cycle in centralized animal housing facility programme managed by Research Animal Resources (RAR) at the Johns Hopkins University. All animal procedures were in strict accordance with the National Institutes of Health Guide for the Care and Use of Laboratory Animals and were approved by the Johns Hopkins University Animal Care and Use Committee.

### Immunoblots and antibodies

Immediately after killing, mouse brains were dissected^63^ in the specific brain regions olfactory bulb, hippocampus, striatum, cortex, midbrain, brain stem, cerebellum and the rest. Proteins of each region were extracted with RIPA buffer (10 mM Tris-Cl (pH 8.0), 1 mM EDTA, 0.5 mM EGTA, 1% Triton X-100, 0.1% sodium deoxycholate, 0.1% SDS and 140 mM NaCl) containing 1 × complete protease inhibitor cocktail (Roche, Indianapolis, IN). The protein concentrations in the supernatants were determined by the BCA method (Pierce Chemical Co., Rockford, IL) and equal amounts of protein lysates (∼20 μg per lane) resolved on 4–12% Bis-Tris SDS–PAGE gels with MES running buffer, then transferred to polyvinylidene difluoride (Invitrogen, Carlsbad, CA) membranes, and probed with following antibodies: anti-human tau polyclonal antiserum KJ9A (1:5,000; A0024, Dako Cooperation, Carpinteria, CA); Purified anti-Tau 316–355 monoclonal antibody 77G7 (1:1,000; BioLegend, San Diego, CA); rabbit anti-APP C-terminal (1:2,000; AB5352, Chemicon); monoclonal antibody against synaptophysin (1:1,000; AB8049, Abcam, Cambridge, MA); rabbit anti-GAPDH antiserum (1:5,000; G9545, Sigma); and monoclonal anti-β-tubulin III antiserum (1:10,000; T2200, Sigma). Immunoblots were developed using enhanced chemiluminescence method (Millipore Corp., MA).

### Histology and immunohistochemical analysis

For the histological and immunohistochemical analysis mice were anaesthetised and killed by decapitation at different ages^64^; brains were removed and weighed. Hemibrains were fixed by submerging into 4% paraformaldehyde (PFA) in PBS, embedded into the paraffin, sectioned in sagittal plane and processed. For histological analyses, 10 μm brain sections were stained with hematoxylin and eosin or Cresyl violet. Hirano silver stain utilized Hirano's modification of the Bielschowsky method^65^,^66^. Briefly, 200 ml of ammonium hydroxide were evaporated under vacuum for 30 min. Sections were deparaffinized for 10 min and placed in 20% sliver solution for 30 min. After a rinse in dH_2_O, slides were transferred to ammoniacal solution (200 ml 20% silver nitrate solution with drops of evaporated ammonium hydroxide) for 15 min. Slides were washed in dH_2_O with four drops of ammonium hydroxide, transferred to ammoniacal solutions with three drops of developer, rinsed in dH_2_O and fixed in 1% sodium thiosulfate for 1 min. Finally the slides were washed with dH_2_O, dehydrated, cleared and mounted.

Gallyas silver staining was modified from Braak^67^ and performed as follows. Slides were deparaffinized and dipped in 5% periodic acid. After a brief wash with water, the slides were incubated for 1 min in silver iodide solution (290 ml dH_2_O, 12 g sodium hydroxide, 30 g potassium iodide, 10 ml 1%silver nitrate). After a wash in 0.5% acetic acid (2 × 5 min), slides were rinsed with dH_2_O and developed for 10–20 min in physical developer solution. After a 5 min wash in 0.5% acetic acid and dH_2_O, slides were incubated in 0.1% gold chloride for 5 min. Next, the slides were rinsed in dH_2_O and fixed in 1% sodium thiosulfate for 5 min. For counterstaining, the slides were dipped in 0.1% nuclear fast red for 2 min before getting washed in tap water, dehydrated and mounted.

The Congo Red stain was performed as followed. Slides were deparaffinized, stained in congo red solution (50 ml 80% alcohol, 0.15 g Congo red, 0.15 g sodium chloride, 0.5 ml 1% sodium hydroxide) for 10 min and rinsed in dH_2_O. The stain was differentiated by dipping the slides 5–10 times in alkaline alcohol solution (100 ml 50% alcohol, 1 ml 1% sodium hydroxide), rinsed in water, dehydrated and mounted.

For the Thioflavin-T staining slides were deparaffinized and hydrated. Slides were incubated in 0.25% potassium permanganate solution for 5 min, transferred to 1% potassium-diulfate and oxcalic acid for 5 min and incubated in 0.02% Thioflavin-T for 8 min. Slides were dehydrated and mounted with aqueous mounting medium for fluorescence.

The area size of hippocampus, cortex and cerebellum in sagittal sections at 2 mm from the midline of the brains were measured by the ImageJ program. Semiquntitative score of Tau tangles was evaluated in low power ( × 200) microscope field as described previously^68^. Score was indicated as: 0, no tangle; 1, 1–5 tangles per field; 2, 6–15 tangles per field; and 3, >15 tangles per field.

For immunohistochemical analysis, antigen retrieval was performed with 10 mM citrate buffer (pH 6.0) for an efficient epitope exposure to the antibodies; endogenous peroxidase was quenched by treating the paraffin sections with 0.3% H_2_O_2_; and nonspecific binding of antibodies was eliminated using blocking buffer (10% normal goat serum in PBS with 0.3% Triton-X) for 1 h at room temperature. The primary antibody prepared in blocking buffer was applied for overnight at 4 ^o^C, followed by a secondary antibody for 30 min incubation at room temperature. For the secondary antibody and avidin-biotinylated peroxidase system, we used the Vectastain Universal Elite ABC kit (Vector Laboratories). Brain sections were stained with: antiserum against Aβ peptides 6E10 (1:1,000; SIG-39300, Covance); antiserum against pan-tau antibody K9JA (1:1,000; A0024, Dako Cooperation, Carpinteria, CA); mouse monoclonal antiserum against phosphorylated tau CP13 (1:1,000; Tau pS202) and PHF-1 (1:1,000, Tau pS396/S404) (gifts of P. Davis, Albert Einstein College of Medicine); rabbit antiserum against phosphorylated T231(1:1,000; 44746G, Invitrogen, Carlsbad, CA), phosphorylated S262 (1:1,000; 44705G, Invitrogen, Carlsbad, CA) phosphorylated S396 (1:1,000; 44752, Invitrogen, Carlsbad, CA) and S422 of tau (1:2,000; 44764G, Invitrogen, Carlsbad, CA); ubiquitin, Microtubule-associated protein 2 (Map2, 1:1,000; AB5622, Millipore), phosphorylated neurofilament (Smi31, 1:1,000; BioLegend, San Diego, CA), neurofilaments in axons (Smi312, 1:1,000; BioLegend, San Diego, CA), Neurofilament triplet H protein (NF-H; 1:1,000, Sigma, Saint Louis, MO, USA), polyclonal antiserum against GFAP (Z0334, Dako Cooperation, Carpinteria, CA); polyclonal antiserum against microglial (IBA1, CP290, Biocare Medical, CA), and monoclonal antibody against NeuN (MAB377, Millipore). Sections for immunofluorescence were examined under a Zeiss LSM 510 laser scanning fluorescence confocal microscope. Z-stack projections were made from serial scanning every 1 μm to reconstruct the Aβ plaques.

### Data analysis

All data were analysed statistically by unpaired Student's two-tailed *t*-test or one-way analysis of variance with Tukey's correction for multiple comparisons using GraphPad Prism version 6.0 f for Mac, GraphPad Software, La Jolla, CA, USA. In all tests, values of *P*<0.05 were considered to indicate significance.

### Data availability

The authors declare that all data supporting the findings of this study are available within the article and its Supplementary Information files or from the authors on request.

## Additional information

**How to cite this article:** Li, T. *et al*. The neuritic plaque facilitates pathological conversion of tau in an Alzheimer's disease mouse model. *Nat. Commun.* 7:12082 doi: 10.1038/ncomms12082 (2016).

## Supplementary Material

Supplementary Information Supplementary Figures 1 – 3

## References

[b1] Holtzman D. M., Morris J. C. & Goate A. M. Alzheimer's disease: the challenge of the second century. Sci. Transl. Med. 3, 77sr1 (2011). 21471435 10.1126/scitranslmed.3002369 PMC3130546

[b2] Hardy J. & Selkoe D. J. The amyloid hypothesis of Alzheimer's disease: progress and problems on the road to therapeutics. Science 297, 353–356 (2002). 12130773 10.1126/science.1072994

[b3] Selkoe D. & Kopan R. Notch and Presenilin: regulated intramembrane proteolysis links development and degeneration. Annu. Rev. Neurosci. 26, 565–597 (2003). 12730322 10.1146/annurev.neuro.26.041002.131334

[b4] Lee V. M., Goedert M. & Trojanowski J. Q. Neurodegenerative tauopathies. Annu. Rev. Neurosci. 24, 1121–1159 (2001). 11520930 10.1146/annurev.neuro.24.1.1121

[b5] Ballatore C., Lee V. M. Y. & Trojanowski J. Q. Tau-mediated neurodegeneration in Alzheimer's disease and related disorders. Nat. Rev. Neurosci. 8, 663–672 (2007). 17684513 10.1038/nrn2194

[b6] Bertram L. & Tanzi R. E. Thirty years of Alzheimer's disease genetics: the implications of systematic meta-analyses. Nat. Rev. Neurosci. 9, 768–778 (2008). 18802446 10.1038/nrn2494

[b7] Bertram L., Lill C. M. & Tanzi R. E. The genetics of Alzheimer disease: back to the future. Neuron 68, 270–281 (2010). 20955934 10.1016/j.neuron.2010.10.013

[b8] Hardy J. A. & Higgins G. A. Alzheimer's disease: the amyloid cascade hypothesis. Science 256, 184–185 (1992). 1566067 10.1126/science.1566067

[b9] Gotz J., Chen F., Van Dorpe J. & Nitsch R. M. Formation of neurofibrillary tangles in P3011 tau transgenic mice induced by Abeta fibrils. Science 293, 1491–1495 (2001). 11520988 10.1126/science.1062097

[b10] Lewis J. . Enhanced neurofibrillary degeneration in transgenic mice expressing mutat tau and APP. Science 293, 1487–1491 (2001). 11520987 10.1126/science.1058189

[b11] Oddo S., Billings L., Kesslak J. P., Cribbs D. H. & LaFerla F. M. Abeta immunotherapy leads to clearance of early, but not late, hyperphosphorylated tau aggregates via the proteasome. Neuron 43, 321–332 (2004). 15294141 10.1016/j.neuron.2004.07.003

[b12] Oddo S. . Blocking Aβ42 accumulation delays the onset and progression of tau pathology via the C terminus of heat shock protein 70-interacting protein: a mechanistic link between alpha beta and tau pathology. J. Neurosci. 28, 12163–12175 (2008). 19020010 10.1523/JNEUROSCI.2464-08.2008 PMC6671718

[b13] Tseng B. P., Green K. N., Chan J. L., Blurton-Jones M. & LaFerla F. M. Aß inhibits the proteasome and enhances amyloid and tau accumulation. Neurobiol. Aging 29, 1607–1618 (2008). 17544172 10.1016/j.neurobiolaging.2007.04.014 PMC2664168

[b14] Goate A. . Segregation of a missense mutation in the amyloid precursor protein gene with familial Alzheimer's disease. Nature 349, 704–706 (1991). 1671712 10.1038/349704a0

[b15] Sherrington R. . Cloning of a gene bearing missense mutations in early-onset familial Alzheimer's disease. Nature 375, 754–760 (1995). 7596406 10.1038/375754a0

[b16] Strittmatter W. J. . Apolipoprotein E: high-avidity binding to b-amyloid and increased frequency of type 4 allele in late-onset familial Alzheimer disease. Proc. Natl Acad. Sci. USA 90, 1977–1981 (1993). 8446617 10.1073/pnas.90.5.1977 PMC46003

[b17] DeMattos R. B. . ApoE and clusterin cooperatively suppress Aβ levels and deposition. Evidence that ApoE regulates extracellular Aβ metabolism *in vivo*. Neuron 41, 193–202 (2004). 14741101 10.1016/s0896-6273(03)00850-x

[b18] Bu G. Apolipoprotein E and its receptors in Alzheimer's disease: pathways, pathogenesis and therapy. Nat. Rev. Neurosci. 10, 333–344 (2009). 19339974 10.1038/nrn2620 PMC2908393

[b19] Kim J., Basak J. M. & Holtzman D. M. The role of apolipoprotein E in Alzheimer's disease. Neuron 63, 287–303 (2009). 19679070 10.1016/j.neuron.2009.06.026 PMC3044446

[b20] Guerreiro R. . TREM2 variants in Alzheimer's disease. N. Engl. J. Med. 368, 117–127 (2013). 23150934 10.1056/NEJMoa1211851 PMC3631573

[b21] Jonsson T. . Variant of TREM2 associated with the risk of Alzheimer's disease. N. Engl. J. Med. 368, 107–116 (2013). 23150908 10.1056/NEJMoa1211103 PMC3677583

[b22] Crystal H. A. . Clinico-pathologic studies in dementia: nondemented subjects with pathologically confirmed Alzheimer's disease. Neurology 38, 1682–1687 (1988). 3185902 10.1212/wnl.38.11.1682

[b23] Katzman R. . Clinical, pathological, and neurochemical changes in dementia: a subgroup with preserved mental status and numerous neocortical plaques. Ann. Neurol. 23, 138–144 (1988). 2897823 10.1002/ana.410230206

[b24] Price J. L. & Morris J. C. Tangles and plaques in nondemented aging and ‘preclinical' Alzheimer's disease. Ann. Neurol. 45, 358–368 (1999). 10072051 10.1002/1531-8249(199903)45:3<358::aid-ana12>3.0.co;2-x

[b25] Arriagada P. V., Growdon J. H., Hedley-Whyte E. T. & Hyman B. T. Neurofibrillary tangles but not senile plaques parallel duration and severity of Alzheimer's disease. Neurology 42, 631–639 (1992). 1549228 10.1212/wnl.42.3.631

[b26] Arriagada P. V., Marzloff K. & Hyman B. T. Distribution of Alzheimer-type pathologic changes in nondemented elderly individuals matches the pattern in Alzheimer's disease. Neurology 42, 1681–1688 (1992). 1307688 10.1212/wnl.42.9.1681

[b27] Gomez-Isla T. . Neuronal loss correlates with but exceeds neurofibrillary tangles in Alzheimer's disease. Ann. Neurol. 41, 17–24 (1997). 9005861 10.1002/ana.410410106

[b28] Price D., Savonenko A. V., Li T., Lee M. K. & Wong P. in Alzheimer Disease: Protein Misfolding, Model Systems, and Experimental Therapeutics in Protein Misfolding Diseases eds Ramirez-Alvarado M., Kelly J. W., Dobson C. M. 233–258John Wiley & Sons, Inc. (2010).

[b29] Gotz J. & Ittner L. M. Animal models of Alzheimer's disease and frontotemporal dementia. Nat. Rev. Neurosci. 9, 532–544 (2008). 18568014 10.1038/nrn2420

[b30] LaFerla F. M. & Green K. N. Animal models of Alzheimer disease. Cold Spring Harb. Perspect. Med. 2, a006320 (2012). 23002015 10.1101/cshperspect.a006320 PMC3543097

[b31] Bales K. R. The value and limitations of transgenic mouse models used in drug discovery for Alzheimer's disease: an update. Expert. Opin. Drug Discov. 7, 281–297 (2012). 22458501 10.1517/17460441.2012.666234

[b32] Platt T. L., Reeves V. L. & Murphy M. P. Transgenic models of Alzheimer's disease: better utilization of existing models through viral transgenesis. Biochim. Biophys. Acta 1832, 1437–1448 (2013). 23619198 10.1016/j.bbadis.2013.04.017 PMC3690778

[b33] Kitazawa M., Medeiros R. & LaFerla F. M. Transgenic mouse models of Alzheimer disease: developing a better model as a tool for therapeutic interventions. Curr. Pharm. Des. 18, 1131–1147 (2012). 22288400 10.2174/138161212799315786 PMC4437619

[b34] Oddo S. . Triple-transgenic model of Alzheimer's disease with plaques and tangles: intracellular Aβ and synaptic dysfunction. Neuron 39, 409–421 (2003). 12895417 10.1016/s0896-6273(03)00434-3

[b35] Lewis J. . Neurofibrillary tangles, amyotrophy and progressive disturbance in mice expressing mutant (P301L) tau protein. Nat. Genet. 25, 402–405 (2000). 10932182 10.1038/78078

[b36] Yoshiyama Y. . Synapse loss and microglial activation precede tangles in a P301S tauopathy mouse mode (vol 53, pg 337, 2007). Neuron 54, 343–344 (2007). 10.1016/j.neuron.2007.01.010 17270732

[b37] SantaCruz K. . Tau suppression in a neurodegenerative mouse model improves memory function. Science 309, 476–481 (2005). 16020737 10.1126/science.1113694 PMC1574647

[b38] Hsiao K. . Correlative memory deficts, Aβ elevation and amyloid plaques in transgenic mice. Science 274, 99–102 (1996). 8810256 10.1126/science.274.5284.99

[b39] Oakley H. . Intraneuronal β-amyloid aggregates, neurodegeneration, and neuron loss in transgenic mice with five familial Alzheimer's disease mutations: potential factors in amyloid plaque formation. J. Neurosci. 26, 10129–10140 (2006). 17021169 10.1523/JNEUROSCI.1202-06.2006 PMC6674618

[b40] Sturchler-Pierrat C. . Two amyloid precursor protein transgenic mouse models with Alzheimer disease-like pathology. Proc. Natl Acad. Sci. USA 94, 13287–13292 (1997). 9371838 10.1073/pnas.94.24.13287 PMC24301

[b41] Duff K. . Characterization of pathology in transgenic mice over-expressing human genomic and cDNA tau transgenes. Neurobiol. Dis. 7, 87–98 (2000). 10783293 10.1006/nbdi.1999.0279

[b42] Winton M. J. . Intraneuronal APP, not free Aβ peptides in 3xTg-AD mice: implications for tau versus Aβ-mediated Alzheimer neurodegeneration. J. Neurosci. 31, 7691–7699 (2011). 21613482 10.1523/JNEUROSCI.6637-10.2011 PMC3118598

[b43] Sanders D. W. . Distinct tau prion strains propagate in cells and mice and define different tauopathies. Neuron 82, 1271–1288 (2014). 24857020 10.1016/j.neuron.2014.04.047 PMC4171396

[b44] Hutton M. . Association of missense and 5'-splice-site mutations in tau with the inherited dementia FTDP-17. Nature 393, 702–705 (1998). 9641683 10.1038/31508

[b45] Wang Y. P., Biernat J., Pickhardt M., Mandelkow E. & Mandelkow E. M. Stepwise proteolysis liberates tau fragments that nucleate the Alzheimer-like aggregation of full-length tau in a neuronal cell model. Proc. Natl Acad. Sci. USA 104, 10252–10257 (2007). 17535890 10.1073/pnas.0703676104 PMC1891218

[b46] Irwin D. J. . Acetylated tau, a novel pathological signature in Alzheimer's disease and other tauopathies. Brain 135, 807–818 (2012). 22366796 10.1093/brain/aws013 PMC3286338

[b47] Cohen T. J. . The acetylation of tau inhibits its function and promotes pathological tau aggregation. Nat. Commun. 2, 252 (2011). 21427723 10.1038/ncomms1255 PMC3120096

[b48] Jankowsky J. L. . Persistent amyloidosis following suppression of Aβ production in a transgenic model of Alzheimer disease. PLoS. Med. 2, e355 (2005). 16279840 10.1371/journal.pmed.0020355 PMC1283364

[b49] Jankowsky J. L., Xu G., Fromholt D., Gonzales V. & Borchelt D. R. Environmental enrichment exacerbates amyloid plaque formation in a transgenic mouse model of Alzheimer disease. J Neuropathol. Exp. Neurol 62, 1220–1227 (2003). 14692698 10.1093/jnen/62.12.1220

[b50] Mocanu M. M. . The potential for beta-structure in the repeat domain of Tau protein determines aggregation, synaptic decay, neuronal loss, and coassembly with endogenous Tau in inducible mouse models of tauopathy. J. Neurosci. 28, 737–748 (2008). 18199773 10.1523/JNEUROSCI.2824-07.2008 PMC6670355

[b51] Harold D. . Genome-wide association study identifies variants at CLU and PICALM associated with Alzheimer's disease. Nat. Genet. 41, 1088–1093 (2009). 19734902 10.1038/ng.440 PMC2845877

[b52] Lambert J. C. . Genome-wide association study identifies variants at CLU and CR1 associated with Alzheimer's disease. Nat. Genet. 41, 1094–1099 (2009). 19734903 10.1038/ng.439

[b53] Hollingworth P. . Common variants at ABCA7, MS4A6A/MS4A4E, EPHA1, CD33 and CD2AP are associated with Alzheimer's disease. Nat. Genet. 43, 429–435 (2011). 21460840 10.1038/ng.803 PMC3084173

[b54] Naj A. C. . Common variants at MS4A4/MS4A6E, CD2AP, CD33 and EPHA1 are associated with late-ons*et al*zheimer's disease. Nat. Genet. 43, 436–441 (2011). 21460841 10.1038/ng.801 PMC3090745

[b55] Jack C. R. Jr & Holtzman D. M. Biomarker modeling of Alzheimer's disease. Neuron 80, 1347–1358 (2013). 24360540 10.1016/j.neuron.2013.12.003 PMC3928967

[b56] Jack C. R. Jr . Tracking pathophysiological processes in Alzheimer's disease: an updated hypothetical model of dynamic biomarkers. Lancet Neurol. 12, 207–216 (2013). 23332364 10.1016/S1474-4422(12)70291-0 PMC3622225

[b57] Choi S. H. . A three-dimensional human neural cell culture model of Alzheimer's disease. Nature (2014). 10.1038/nature13800 PMC4366007 25307057

[b58] Golde T. E., Schneider L. S. & Koo E. H. Anti-Aβ therapeutics in Alzheimer's disease: the need for a paradigm shift. Neuron 69, 203–213 (2011). 21262461 10.1016/j.neuron.2011.01.002 PMC3058906

[b59] Sperling R. A., Jack C. R. Jr & Aisen P. S. Testing the right target and right drug at the right stage. Sci. Transl. Med. 30, *111cm33* (2011). 10.1126/scitranslmed.3002609 PMC3752906 22133718

[b60] Salloway S., Sperling R. & Brashear H. R. Phase 3 trials of solanezumab and bapineuzumab for Alzheimer's disease. N. Engl. J. Med. 370, 1460 (2014). 24724181 10.1056/NEJMc1402193

[b61] Doody R. S., Farlow M. & Aisen P. S. Phase 3 trials of solanezumab and bapineuzumab for Alzheimer's disease. N. Engl. J. Med. 370, 1460 (2014). 24716687 10.1056/NEJMc1402193

[b62] Mayford M. . Control of memory formation through regulated expression of a CaMKII transgene. Science 274, 1678–1683 (1996). 8939850 10.1126/science.274.5293.1678

[b63] Li T., Ma G., Cai H., Price D. L. & Wong P. C. Nicastrin is required for assembly of presenilin/gamma -secretase complexes to mediate notch signaling and for processing and trafficking of β -amyloid precursor protein in mammals. J. Neurosci. 23, 3272–3277 (2003). 12716934 10.1523/JNEUROSCI.23-08-03272.2003 PMC6742329

[b64] Li T. . Moderate reduction of gamma-secretase attenuates amyloid burden and limits mechanism-based liabilities. J. Neurosci. 27, 10849–10859 (2007). 17913918 10.1523/JNEUROSCI.2152-07.2007 PMC6672827

[b65] Yamamoto T. & Hirano, A. A comparative study of modified Bielschowsky, Bodian and thioflavin S stains on Alzheimer's neurofibrillary tangles. Neuropathol. Appl. Neurobiol. 12, 3–9 (1986). 2422580 10.1111/j.1365-2990.1986.tb00677.x

[b66] Borchelt D. R. . Familial Alzheimer's disease-linked presenilin 1 variants elevate Aβ1-42/1-40 ratio *in vitro* and *in vivo*. Neuron 17, 1005–1013 (1996). 8938131 10.1016/s0896-6273(00)80230-5

[b67] Braak H. & Braak E. Neuropathological staging of Alzheimer-related changes. Acta Neuropathol. 82, 239–259 (1991). 1759558 10.1007/BF00308809

[b68] Mirra S. S., Hart M. H. & Terry R. D. Making the diagnosis of Alzheimer's disease. A primer for practicing pathologists. Arch. Pathol. Lab. Med. 117, 132–144 (1993). 8427562

