# Supplementary material for: The neuritic plaque facilitates pathological conversion of tau in an Alzheimer's disease mouse model
Source: Nat Commun. 2016 Jul 4;7:12082. doi: 10.1038/ncomms12082 (PMC4932197; doi:10.1038/ncomms12082)
Supplement: Supplementary Information — Supplementary Figures 1 – 3 [file ncomms12082-s1.pdf]

Supplementary

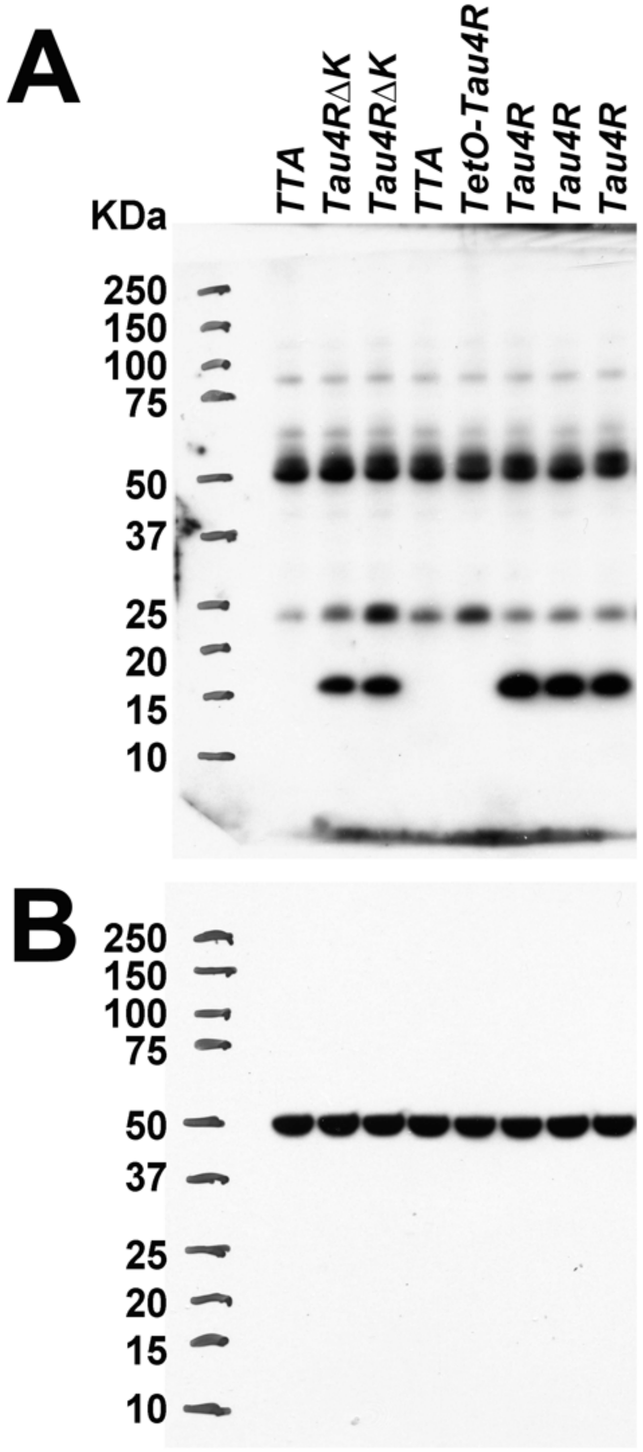

Supplementary Fig. 1 Protein blot of *Tau4R* and *Tau4RDK* mice

A. Protein blot using 77G7 antibody that recognized the repeat domain of tau showed the presence of exogenous (~16 kDa) TauRD or TauRDΔK280 and endogenous tau protein from brain lysates of tau transgenic (*Tau4R* and *Tau4RΔK*) mice. The expression level of exogenous tau in *Tau4R* and *Tau4RΔK* mice was similar to that of non-transgenic mice.

B. The same blot in panel A was re-probed with antiserum against β-tubulin III.

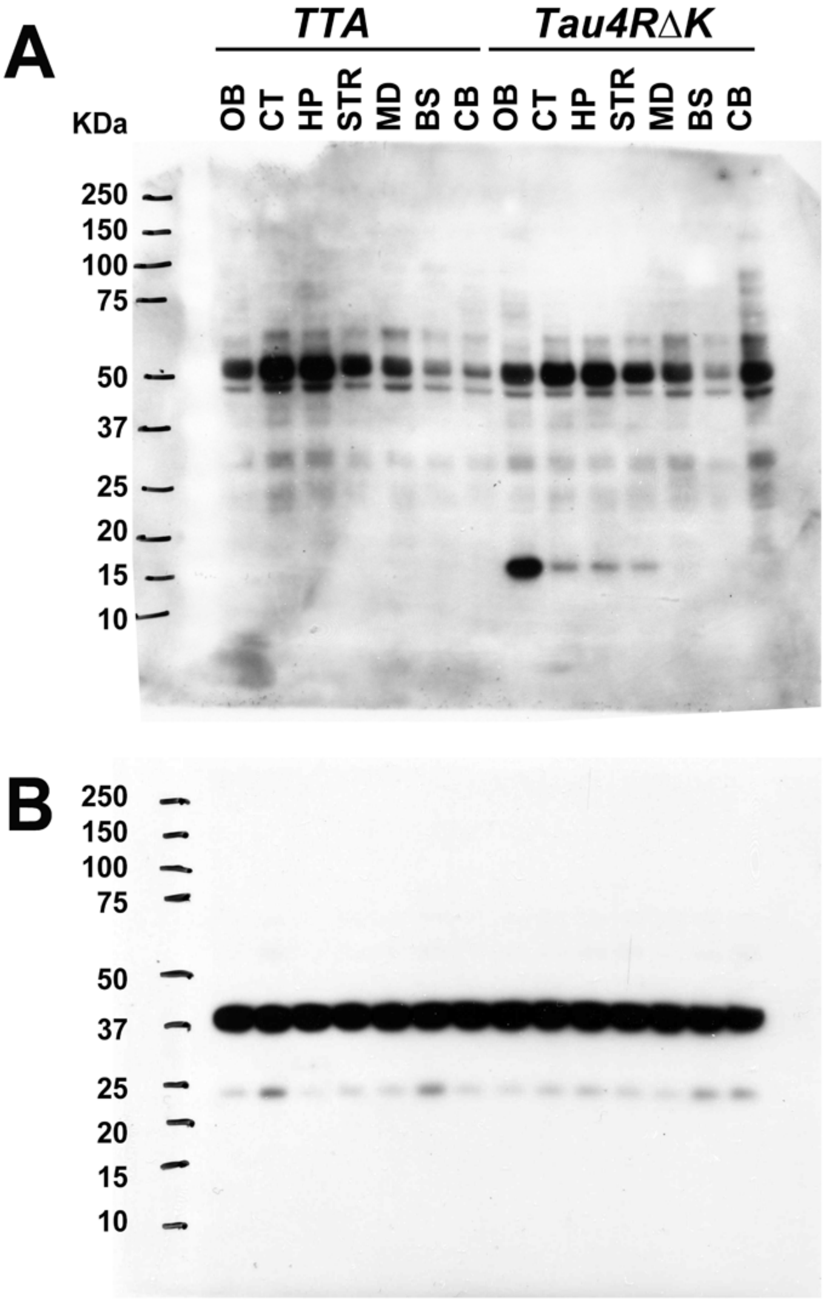

Supplementary Figure 2. Protein blot of different region of *Tau4RΔK* mice brain

A. Total protein was extracted from different brain regions: olfactory bulb (OB), cortex (CT), hippocampus (HP), striatum (STR), midbrain (MB), brainstem (BS), cerebellum (CE) of *TTA* and *Tau4RΔK* mice. Human tau fragment (~16 kDa) detected using anti-human tau polyclonal antiserum KJ9A was only seen in frontal

region of the brain (OB, CT, HP, and STR), but not in midbrain, brain stem or cerebellum.

B. The same blot in panel A was re-probed using an antibody against synaptophysin.

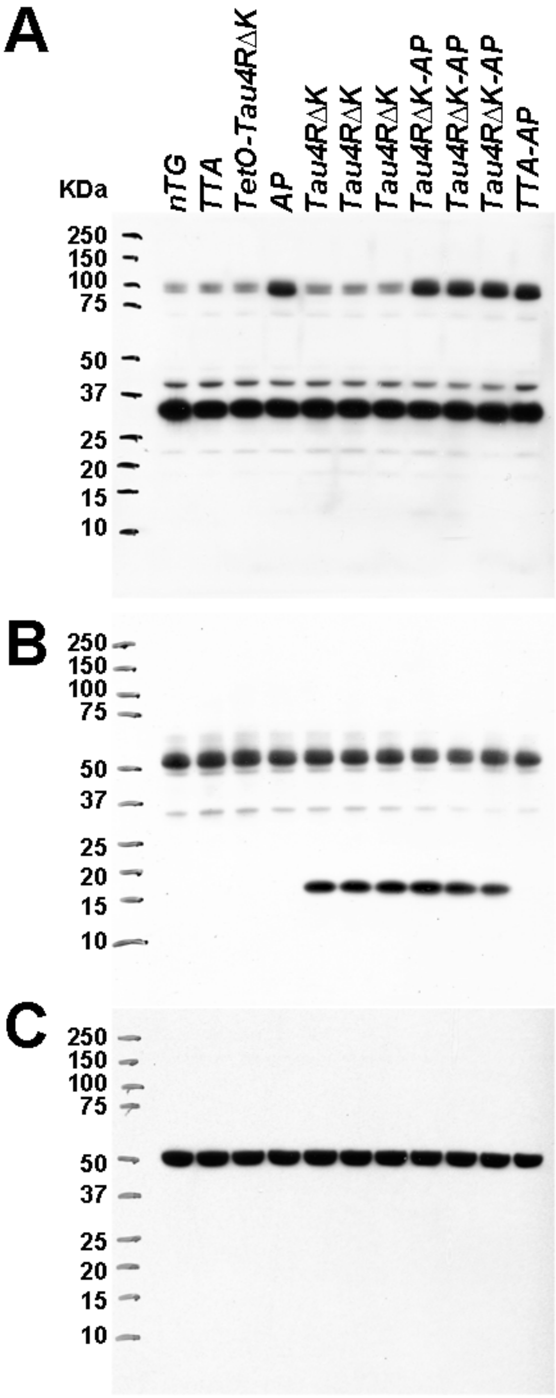

Supplementary Fig. 3 Protein blot of *Tau4RDK-AP* mice

A. Protein blot analysis of APP in brain lysates of 3 month-old female *TTA, tetO-tauRDΔK, AP*, *TTA-AP*, *Tau4RΔK, Tau4RΔK-AP* and *nTG* mice using antisera CT15.

B. The same blot in panel A was re-probed using antisera K9JA.

C. The same blot in panel A was re-probed with antiserum against β-tubulin III.
